# DASH Diet and Preeclampsia Prevention: A Literature Review

**DOI:** 10.3390/nu17122025

**Published:** 2025-06-17

**Authors:** Dimitris Baroutis, Eleni Katsianou, Diamantis Athanasiou, Aikaterini-Gavriela Giannakaki, Panagiotis Antsaklis, Marianna Theodora, George Daskalakis, Makarios Eleftheriades

**Affiliations:** 1 1st Department of Obstetrics & Gynecology, Alexandra Hospital, National and Kapodistrian University of Athens, 11528 Athens, Greece; diamathan16@gmail.com (D.A.); giannakaki.katerina@gmail.com (A.-G.G.); panosant@gmail.com (P.A.); martheodr@gmail.com (M.T.); gdaskalakis@yahoo.com (G.D.); 2 2nd Department of Obstetrics & Gynecology, Aretaieio Hospital, National and Kapodistrian University of Athens, 11528 Athens, Greece; elkatsianou@gmail.com (E.K.); makarios@hotmail.co.uk (M.E.)

**Keywords:** DASH diet, preeclampsia, pregnancy, hypertensive disorders of pregnancy, prevention, nutrition, cardiovascular health

## Abstract

The Dietary Approaches to Stop Hypertension (DASH) diet, characterized by high consumption of fruits, vegetables, whole grains, low-fat dairy products, and limited intake of saturated fats, cholesterol, and refined sugars, has been suggested to reduce the risk of preeclampsia. This narrative review aimed to summarize and synthesize the evidence regarding the role of the DASH diet in preeclampsia prevention. PubMed/MEDLINE, Embase, Google Scholar, ScienceDirect, Scopus, and Web of Science databases were searched to identify relevant studies. Multiple observational and intervention studies examining DASH diet adherence and preeclampsia outcomes were included. Higher adherence to the DASH diet was associated with an approximately 35–45% reduced risk of preeclampsia in observational studies. Intervention trials in high-risk populations demonstrated improved blood pressure control and potential reductions in preeclampsia incidence. The DASH diet appears to exert protective effects through multiple mechanisms, including improved blood pressure regulation, enhanced antioxidant defense, reduced inflammation, and improved endothelial function. The heterogeneity in study designs, DASH diet assessment methods, and intervention protocols limited the strength of conclusions. Evidence for the effects of greater adherence to the DASH diet on preeclampsia prevention is promising but requires confirmation through larger randomized controlled trials. Future research should focus on standardized DASH diet assessment methods, optimal timing and duration of dietary intervention, and exploration of potential synergies with other preventive strategies.

## 1. Introduction

Hypertensive disorders of pregnancy, specifically preeclampsia, constitute leading contributors to adverse maternal-fetal outcomes worldwide, with global incidence patterns affecting 2–5% of gestations and representing primary determinants of maternal mortality [1,2]. This pregnancy-specific hypertensive disorder is characterized by new-onset hypertension after 20 weeks of gestation accompanied by proteinuria and/or maternal organ dysfunction [3,4]. Despite decades of research and advances in prenatal care, preeclampsia remains a leading cause of maternal mortality, accounting for approximately 46,000 maternal deaths and 500,000 perinatal deaths annually [1,2]. The disease burden is disproportionately borne by women in low- and middle-income countries or those otherwise disadvantaged, highlighting significant health disparities in maternal care worldwide [5,6].

The global incidence of preeclampsia has remained relatively stable over time, though regional variations exist. Incidence rates range from as low as 1% in some European countries to over 7% in certain developing regions. This geographic variability reflects differences in risk factors, healthcare access, diagnostic criteria, and possibly dietary patterns across populations [2,5]. In the United States, preeclampsia affects approximately 3–5% of pregnancies and is responsible for 15% of maternal deaths and 25% of medically indicated preterm deliveries [7,8].

### 1.1. Pathophysiology of Preeclampsia

Preeclamptic pathogenesis encompasses sequential vascular dysfunction phases, initiating with defective trophoblast invasion during early gestation and progressing to maternal endothelial activation that culminates in characteristic clinical manifestations [9,10]. The fundamental pathogenic mechanism involves insufficient trophoblastic penetration of maternal spiral vasculature throughout placental development. During normal gestational progression, trophoblastic cells infiltrate and restructure maternal spiral arteries, converting high-resistance, limited-capacity vessels into low-resistance, high-capacity conduits adequate for meeting escalating fetal metabolic demands. In preeclamptic conditions, this vascular remodeling process becomes compromised, maintaining narrow, high-resistance arterial architecture [9,10].

Such deficient placental establishment triggers placental oxygen deprivation, tissue ischemia, oxidative cellular damage, and liberation of multiple factors into maternal systemic circulation, encompassing anti-angiogenic mediators, pro-inflammatory cytokines, syncytiotrophoblast cellular fragments, and circulating fetal genetic material [9,10,11]. Within these mechanisms, disrupted equilibrium between pro-angiogenic mediators (including placental growth factor, PlGF) and anti-angiogenic substances (including soluble fms-like tyrosine kinase-1, sFlt-1, and soluble endoglin, sEng) assumes critical importance. Increased sFlt-1 concentrations bind circulating PlGF and vascular endothelial growth factor (VEGF), blocking their endothelial cell receptor interactions and triggering endothelial dysfunction [12,13].

This endothelial impairment constitutes the primary pathophysiological foundation of maternal syndrome manifestation, affecting multiple organ systems including renal, hepatic, and central nervous system function [3,4,7,9,11]. The consequent systemic inflammatory activation, enhanced vascular responsiveness, and coagulation cascade stimulation generate the clinical presentations characteristic of preeclampsia [11]. Contemporary research has additionally identified supplementary contributors to preeclamptic pathogenesis, encompassing immunological maladaptation, hereditary susceptibility, excessive complement system activation, and renin–angiotensin–aldosterone system modifications [9,10,11].

### 1.2. Classification and Clinical Manifestations

Preeclampsia has been traditionally classified based on the timing of onset, with important prognostic and pathophysiological implications [9,10,11,14]. Early-onset preeclampsia (developing before 34 weeks’ gestation) is more often associated with inadequate placentation, severe maternal and fetal complications, and a hemodynamic profile of low cardiac output and high peripheral vascular resistance [14]. This subtype is more likely to be associated with fetal growth restriction, reflecting the significant placental involvement in its pathogenesis. In contrast, late-onset preeclampsia (at or after 34 weeks’ gestation), which accounts for at least 70% of cases, usually presents with normal or even increased birth weight, potentially increased cardiac output, and variable peripheral vascular resistance (either decreased [14] or increased [15]). While much of the literature focuses on preterm preeclampsia, which is associated with a substantially higher risk of maternal and fetal complications than term preeclampsia, the latter accounts for approximately two-thirds of cases and makes a substantial contribution to overall preeclampsia-related morbidity [7,8].

More recently, preeclampsia has been subclassified based on the presence or absence of severe features, which include severe hypertension (systolic blood pressure ≥ 160 mmHg or diastolic blood pressure ≥110 mmHg), severe proteinuria, thrombocytopenia, impaired liver function, renal insufficiency, pulmonary edema, new-onset cerebral or visual disturbances, and uteroplacental insufficiency [3,4]. This classification has important implications for management and prognosis.

The clinical manifestations of preeclampsia are multisystemic and reflect widespread endothelial dysfunction [9,11]. Cardiovascular manifestations primarily involve increased peripheral vascular resistance producing hypertension despite reduced intravascular volume [14]. Pulmonary endothelial activation, neutrophil stimulation, and decreased plasma oncotic pressure elevate pulmonary edema and acute respiratory distress syndrome risk [15]. Severe hypertension, particularly systolic elevation, increases hemorrhagic stroke risk, while hypertension combined with endothelial activation may result in reversible ischemic encephalopathy and eclamptic seizures [14,15].

Renal manifestations most commonly present as proteinuria secondary to glomerular endotheliosis and podocyte injury [7]. Liver involvement can include periportal inflammation, hepatocellular damage, and rarely subcapsular hematoma or hepatic rupture [8]. Hematologic manifestations include thrombocytopenia, hemolysis, and occasionally disseminated intravascular coagulation [14]. Fetal complications encompass growth restriction secondary to placental insufficiency and macrosomia due to uteroplacental mismatch [15]. Additionally, preeclampsia is associated with increased risks of preterm delivery, contributing to neonatal morbidity and mortality [7,8]. Neurological complications in the offspring may include cerebral palsy and encephalopathies, particularly in cases of severe preeclampsia with early delivery, reflecting the complex interplay between maternal vascular dysfunction, placental insufficiency, and fetal hypoxic-ischemic injury [1,2].

### 1.3. Risk Factors and Prediction

Numerous preeclamptic risk factors have been characterized, typically categorized as maternal, paternal, and placental contributors. Maternal biological and social risk determinants encompass specific demographic characteristics (including minority racial or ethnic group membership, age extremes), medical or obstetrical disorder history (including chronic hypertension, pre-existing diabetes, previous preeclampsia, primigravidity), current pregnancy characteristics (including multifetal gestation, assisted reproductive technology conception), physiological abnormalities (including elevated blood pressure, ssity), abnormal laboratory findings (including severe anemia), and ultrasonographic abnormalities (including abnormal uterine artery pulsatility index via Doppler ultrasonography) [3,11].

Preeclamptic risk stratification represents fundamental elements for targeted prevention implementation among vulnerable populations. Conventional screening approaches evaluate clinical risk determinants during early gestational periods, treating them independently and aggregating them without risk level indication or factor enumeration [2]. This methodology demonstrates simplicity but achieves limited detection rates for preterm preeclampsia (approximately 40%) and term preeclampsia (approximately 35%), with positive screening rates approaching 10% [3].

Advanced multivariable modeling systems have been established with enhanced detection capabilities when implemented at 11–13 weeks’ gestation for preterm preeclampsia and 35–36 weeks’ gestation for term preeclampsia [3,5]. The Fetal Medicine Foundation (FMF)’s competing risks model, supported by substantial evidence, incorporates maternal characteristics (including ethnic/racial background and body mass index), blood pressure measurements, uterine artery pulsatility indices, and angiogenic markers [5]. This model identifies approximately 90% of women at 11–13 weeks’ gestation who will develop early preeclampsia and approximately 75% of those developing preterm preeclampsia, maintaining a 10% positive screening rate [3].

### 1.4. Prevention Strategies

The prevention of preeclampsia is a priority in maternal healthcare, considering that definitive treatment requires placental delivery. Current preventive strategies focus on addressing the pathophysiological mechanisms involved in preeclampsia development, including angiogenic imbalance, endothelial activation, oxidative stress, inflammation, and vasoconstriction [11].

#### 1.4.1. Pharmacological Interventions

Multiple pharmacological approaches have undergone extensive investigation, with low-dose aspirin representing the most broadly endorsed intervention for high-risk maternal populations [16,17]. Meta-analytic evaluation of 60 trials encompassing 36,716 women with elevated preeclamptic risk demonstrated that aspirin therapy (50–162 mg daily, typically ≤75 mg daily) achieves dose-dependent preeclamptic risk reduction (relative risk, 0.82; 95% CI, 0.77 to 0.88), while simultaneously reducing serious maternal complications, preterm delivery, small-for-gestational-age infant delivery, and fetal or neonatal mortality [10].

The landmark ASPRE (Combined Multimarker Screening and Randomized Patient Treatment with Aspirin for Evidence-Based Preeclampsia Prevention) investigation demonstrated that aspirin therapy (150 mg daily) administered from 11–13 weeks’ gestation through 36 weeks’ gestation achieved greater than 60% preterm preeclamptic risk reduction (odds ratio, 0.38; 95% CI, 0.20 to 0.74) [16]. Subsequent meta-analytic confirmation established aspirin efficacy in preterm preeclamptic prevention (relative risk, 0.62; 95% CI, 0.45 to 0.87) but not term disease manifestations, contingent upon treatment initiation by 16 weeks’ gestation at minimum doses of 100 mg daily [17].

Calcium supplementation represents an additional effective preventive approach, which is particularly beneficial for women with inadequate dietary calcium consumption. Meta-analytic examination of 30 trials encompassing 20,445 women demonstrated that gestational calcium supplementation reduces preeclamptic risk (relative risk, 0.49; 95% CI, 0.39 to 0.61), with efficacy observed predominantly in women with low baseline calcium intake (<900 mg per day) [18].

Other pharmacological interventions have been investigated, including pravastatin, folic acid, low-molecular-weight heparin, and metformin, but evidence supporting their routine use for preeclampsia prevention remains limited [16,17,18]. Pravastatin, which has lipid-lowering, anti-inflammatory, and pro-angiogenic properties, has shown promise in preliminary studies but requires further investigation in larger trials [17]. Similarly, while some observational studies have suggested a protective effect of folic acid supplementation beyond the recommended dose for neural tube defect prevention, randomized trials have not consistently supported this finding [17,18].

#### 1.4.2. Non-Pharmacological Interventions

Non-pharmacological approaches to preeclampsia prevention have also been explored, with varying levels of evidence. Exercise has been shown to reduce the risk of preeclampsia (odds ratio, 0.59; 95% CI, 0.37 to 0.90) without adverse fetal effects [9]. To achieve these benefits, women must undertake at least 140 min per week of moderate-intensity exercise. The mechanisms by which exercise reduces preeclampsia risk may include improved placental perfusion, enhanced antioxidant defenses, reduced inflammation, and improved endothelial function [9].

Weight management before and during pregnancy represents another potential preventive strategy, given the strong association between obesity and preeclampsia risk [11,12]. However, evidence for the effectiveness of weight loss interventions initiated during pregnancy is limited and potentially concerning, as excessive weight loss may adversely affect fetal growth. Pre-pregnancy weight optimization appears to be a more promising approach [19].

Dietary interventions have gained increasing attention as potential preventive measures for preeclampsia [19]. Diet represents a modifiable risk factor that may influence the pathophysiological mechanisms underlying preeclampsia, including inflammation, oxidative stress, endothelial function, and angiogenic balance. Multiple dietary factors have been associated with preeclampsia risk. A systematic review by Schoenaker et al. found that higher intake of fruits, vegetables, whole grains, and plant-based foods was associated with lower risk of preeclampsia [19]. Conversely, diets high in processed foods, refined sugars, and saturated fats may increase risk. Specific nutrients, including antioxidants, omega-3 fatty acids, vitamin D, and dietary fiber, may also play protective roles [20,21,22,23].

Rather than focusing on individual nutrients, research has increasingly examined dietary patterns, which may better capture the complex interactions among various food components. Among these dietary patterns, the Mediterranean diet has shown promising results in reducing the risk of preeclampsia and other pregnancy complications [24,25], as highlighted in a recent review study which found that higher adherence to the Mediterranean diet was associated with improved clinical pregnancy rates and live birth rates in women undergoing assisted reproductive technology [26].

### 1.5. DASH Diet: Overview and Potential Application in Preeclampsia

Another dietary pattern that has gained significant attention for its cardiovascular benefits and potential role in preeclampsia prevention is the Dietary Approaches to Stop Hypertension (DASH) diet. The DASH diet was originally developed in the 1990s as a dietary intervention specifically designed to reduce blood pressure [27,28]. Given that hypertension is a defining feature of preeclampsia, and considering the shared pathophysiological mechanisms between chronic hypertension and preeclampsia (including endothelial dysfunction, oxidative stress, and inflammation) [7,11,16], the DASH diet has emerged as a promising approach for preeclampsia prevention.

#### 1.5.1. Fundamentals of the DASH Diet

The DASH dietary architecture prioritizes nutrient-dense whole foods—emphasizing fresh produce, unrefined grains, and reduced-fat dairy—while incorporating moderate lean protein portions from poultry, fish, and nuts [29]. This nutritional framework systematically minimizes saturated fats and processed sugars while optimizing essential mineral profiles including potassium, magnesium, calcium, and dietary fiber [30].

Phytoconstituent Profile: DASH dietary components deliver extensive arrays of plant-derived bioactive molecules offering therapeutic potential. Produce sources supply diverse flavonoid compounds (quercetin, catechins, anthocyanins), carotenoid pigments (beta-carotene, lycopene, lutein), and polyphenolic substances (resveratrol, chlorogenic acid) demonstrating robust antioxidant and anti-inflammatory capacity [29,30]. Unrefined grain products supply phenolic acids and lignan compounds, while tree nuts and legumes provide phytosterols alongside additional polyphenolic constituents [29,30]. These bioactive molecules function through coordinated mechanisms targeting oxidative stress reduction, inflammatory cascade modulation, and vascular endothelial enhancement—pathways directly relevant to preeclamptic pathogenesis [7,11,16,29,30].

Specifically, the standard DASH diet recommends:

- 4–5 servings of vegetables per day
- 4–5 servings of fruits per day
- 6–8 servings of whole grains per day
- 2–3 servings of low-fat dairy products per day
- 2 or fewer servings of lean meats, poultry, and fish per day
- 4–5 servings of nuts, seeds, and legumes per week
- Limited intake of fats and sweets
- Sodium restriction (2300 mg per day in the standard DASH diet, 1500 mg per day in the low-sodium DASH diet)

The DASH diet’s effectiveness in lowering blood pressure has been well established in the general population. The original DASH trial demonstrated that the diet could reduce systolic blood pressure by 5.5 mmHg and diastolic blood pressure by 3.0 mmHg in individuals with normal blood pressure, and by 11.4 mmHg and 5.5 mmHg, respectively, in individuals with hypertension [31]. These effects were observed independent of sodium restriction, although combining the DASH diet with reduced sodium intake provided additional blood pressure-lowering benefits [32].

#### 1.5.2. DASH Diet in Women’s Health

The application of the DASH diet in women’s health, particularly during pregnancy, has been an area of growing research interest. Several physiological mechanisms make the DASH diet potentially beneficial during pregnancy:

Mineral Balance: The DASH diet’s high content of calcium, magnesium, and potassium may help maintain normal vascular function during pregnancy. Calcium is essential for vascular smooth muscle contraction and relaxation, while magnesium acts as a natural calcium channel blocker, potentially reducing vascular resistance [33]. Potassium helps maintain fluid balance and may counteract the effects of sodium on blood pressure [34].

Antioxidant Effects: The high fruit and vegetable content of the DASH diet provides abundant antioxidants, including vitamins C and E, carotenoids, and flavonoids. These compounds may help combat the oxidative stress that is implicated in preeclampsia pathogenesis [35,36].

Anti-inflammatory Properties: The DASH diet’s emphasis on whole foods and limitation of processed foods may reduce systemic inflammation. Many components of the diet, including fruits, vegetables, whole grains, and nuts, contain anti-inflammatory compounds that could potentially modulate the inflammatory response associated with preeclampsia [37].

Homocysteine Metabolism: DASH nutritional architecture delivers substantial B-vitamin cofactors including folate (derived from produce sources), pyridoxine (from whole grain and protein sources), and cobalamin (from dairy and lean meat components) that facilitate homocysteine metabolic processing [38,39]. Hyperhomocysteinemia correlates with elevated preeclamptic risk through pathways encompassing vascular endothelial impairment, cellular oxidative damage, and thrombotic activation [40,41]. Through adequate B-vitamin provision, DASH dietary patterns may support optimal homocysteine clearance and mitigate associated cardiovascular risks [42,43].

Endothelial Function: Several components of the DASH diet, including potassium, magnesium, and dietary nitrates from vegetables, may improve endothelial function. Given that endothelial dysfunction is central to preeclampsia pathophysiology, this mechanism could be particularly relevant [44,45,46,47].

## 2. Materials and Methods

This narrative review was conducted to synthesize and analyze the existing literature on the DASH diet and its role in the prevention and management of preeclampsia. While we followed a structured approach to literature search and selection, this review was not conducted according to PRISMA guidelines, as it is not a systematic review or meta-analysis.

### 2.1. Search Strategy

Evidence identification utilized six electronic databases: PubMed/MEDLINE, Embase, Google Scholar, ScienceDirect, Scopus, and Web of Science (Clarivate), with retrieval activities spanning September 2024 through April 2025. The following search algorithm was used:

(Preeclampsia OR Pre-eclampsia OR toxemia OR toxemia OR eclampsia) AND (“DASH diet” OR “Dietary Approaches to Stop Hypertension” OR “DASH pattern” OR “DASH-style diet” OR “DASH dietary pattern”).

Additionally, the ‘snowball literature searching method’ was employed to identify additional relevant sources from the reference lists of selected articles.

### 2.2. Study Selection

The selection of included studies was carried out based on their relevance to the subject in terms of their title and abstract, followed by a full-text examination. The inclusion criteria were:

1. Studies examining the DASH diet as a whole dietary pattern in relation to preeclampsia prevention or risk reduction.
2. Studies focusing on pregnant women or women of reproductive age.
3. Published original research studies, including randomized controlled trials, cohort studies, case-control studies, and cross-sectional studies.
4. Studies including methods for evaluating adherence to the DASH diet.

Exclusion criteria were:

1. Studies focusing only on specific ingredients, vitamins, or trace elements rather than the DASH diet as a whole.
2. Studies focusing only on other pregnancy complications without mentioning preeclampsia or gestational hypertensive disorders.
3. Studies focusing only on blood pressure management in non-pregnant populations without assessing preeclampsia or gestational hypertensive disorders as outcomes.
4. Literature reviews, systematic reviews, and case reports (though these were examined for potential primary sources).

There was no time limit applied, and only English language publications were considered.

### 2.3. Data Extraction and Synthesis

Two reviewers (D.B. & E.K.) independently extracted data from the selected studies using a standardized form. The extracted information included the following: first author, publication year, country, study design, sample size and characteristics, method of evaluating DASH diet adherence, duration of study and follow-up period, main results, and confounding factors considered.

Our review evaluated the association between adherence to the DASH diet before and/or during pregnancy and the risk of developing preeclampsia. We examined both the direct associations with preeclampsia as well as effects on related pathophysiological mechanisms.

### 2.4. Quality Assessment

While a formal quality assessment of included studies was not conducted due to the narrative nature of this review, we critically evaluated each study’s methodology, sample size, and potential biases during our analysis and interpretation of results. Our evaluation considered the following aspects:

- Study design: We assessed whether the design (randomized controlled trial, cohort, case-control, cross-sectional) was appropriate for addressing the research question.
- Sample size: We considered the number of participants in each study.
- DASH diet assessment methods: We evaluated the tools used to measure diet adherence, including the number of food items assessed and whether the tools were validated.
- Outcome measures: We assessed the clarity and relevance of the reported outcomes related to preeclampsia or its pathophysiological mechanisms.
- Confounding factors: We examined which potential confounders were considered in each study.
- Reporting of results: We assessed the clarity and completeness of the reported findings.

These evaluations informed our interpretation of results and discussion of study limitations, allowing us to contextualize the findings within the broader literature on the DASH diet and preeclampsia prevention.

## 3. Results

### 3.1. DASH Diet and Preeclampsia Incidence

Several studies have investigated the relationship between DASH diet adherence and hypertensive disorders of pregnancy, with varying designs, populations, and outcomes. Table 1 summarizes the key characteristics of these studies, providing a comprehensive overview of the current evidence base examining DASH diet and preeclampsia prevention.

In a prospective cohort study involving 16,892 singleton pregnancies among 11,535 women from the Nurses’ Health Study II, Arvizu et al. observed a significant inverse association between DASH diet adherence and preeclampsia risk [48]. After adjusting for multiple confounders, women in the highest quintile of DASH score had a 35% lower risk of preeclampsia compared to those in the lowest quintile (RR: 0.65; 95% CI: 0.48, 0.87; *p*-trend = 0.01) [48].

Cao et al. reported similar findings in a case-control study of 449 preeclampsia cases and 449 controls in China [49]. Participants in the fourth quartile of DASH score were 45% less likely to have preeclampsia than those in the first quartile (crude OR: 0.55; 95% CI: 0.38, 0.80; *p*-trend = 0.001), with the relationship remaining significant after adjusting for multiple confounders (adjusted OR: 0.53; 95% CI: 0.36, 0.78; *p*-trend = 0.001) [49].

In a randomized controlled trial by Belfort et al. examining the effect of the DASH diet on preeclampsia incidence in pregnant women with pre-existing diabetes mellitus, the results showed no statistically significant difference between intervention groups (22.9% in standard diet vs. 12.1% in DASH diet group, *p* = 0.25) [50]. This suggests a potential benefit that did not reach statistical significance in this high-risk population.

Vesco et al. conducted a randomized controlled trial examining the efficacy of a group-based dietary intervention for limiting gestational DASH diet with sodium restriction and at least 30 min of moderate physical activity. Although the primary outcome was gestational weight gain, the study also assessed secondary outcomes including preeclampsia. The incidence of preeclampsia was 10% in the control group compared to 9% in the intervention group, but this difference did not reach statistical significance (*p* = 0.101) (OR: 0.85; 95% CI: 0.24–2.96) [51].

The findings across studies examining DASH diet effects on preeclampsia and hypertensive disorders show varying levels of evidence strength and significance, as summarized in Table 2. While observational studies generally demonstrate strong inverse associations, intervention trial results are more mixed, suggesting potential benefits that warrant further investigation in larger randomized controlled trials.

### 3.2. DASH Diet and Blood Pressure During Pregnancy

Courtney et al. conducted an observational study of 511 women from the ROLO study and found that higher adherence to the DASH dietary pattern throughout pregnancy was associated with significantly lower diastolic blood pressure (DBP) in trimesters 1 (B: −0.70; 95% CI: −1.21 to −0.18; *p* = 0.008) and 3 (B: −0.68; 95% CI: −1.19 to −0.17; *p* = 0.010), as well as lower mean arterial pressure (MAP) in trimesters 1 and 3. For each five-unit increase in overall DASH score, participants had a 0.8 mmHg lower DBP in trimester 1 (*p* = 0.001) and 0.76 mmHg lower DBP in trimester 3 (*p* = 0.05) [52].

In a population-based cohort study among 3414 Dutch women, Wiertsema et al. found that lower maternal DASH score quartiles were associated with a higher mid-pregnancy diastolic blood pressure compared with the highest quartile (*p* < 0.05). A higher maternal DASH score across the full range was significantly associated with a lower mid-pregnancy diastolic blood pressure (difference −0.45 [95% CI: −0.78 to −0.12] mm Hg per SD increase). No associations were present for early- and late-pregnancy diastolic blood pressure or systolic blood pressure throughout pregnancy in this low-risk population [53].

Santos et al. [54] conducted a nutrigenetic trial with 70 pregnant women with pregestational diabetes mellitus, randomized to either traditional or DASH diet groups. Although no significant differences in blood pressure trajectory were found between diet groups, both groups showed normal physiological patterns of blood pressure throughout pregnancy, with increases in systolic and diastolic values only after 32.5 weeks of gestation [54].

### 3.3. DASH Diet and Pregnancy Outcomes

Najafian et al. conducted a randomized controlled trial on 60 pregnant women with gestational or chronic hypertension and found significant improvements in clinical outcomes among women following the DASH diet. After 1 and 2 months of intervention, systolic and diastolic blood pressure in the DASH diet group was significantly lower than the control group (*p* < 0.05). The incidence of preeclampsia (*p* = 0.035), preterm delivery (*p* = 0.020), and placental abruption (*p* = 0.007) was also significantly lower in the DASH diet group [55].

These findings are supported by Jiang et al.’s study examining the efficacy of the DASH diet in 85 pregnant women diagnosed with gestational or chronic hypertension. The incidence of preeclampsia in the control group was 65.9% versus 43.2% in the DASH group (*p* = 0.036), with significant differences also observed in gestational age at delivery and newborn body length between the two groups (*p* < 0.05) [56].

Wiertsema et al. found that compared with the highest maternal DASH score quartile, the lower DASH score quartiles were associated with a higher umbilical artery pulsatility index (*p* ≤ 0.05) in mid and late pregnancy but not with the uterine artery resistance index. This suggests improved fetoplacental vascular function with higher DASH diet adherence, although these benefits did not translate to reductions in gestational hypertensive disorders in this low-risk population [53].

However, not all studies have found significant associations. In a study involving 1760 women in Project Viva, a Boston-area longitudinal cohort, Fulay et al. found that adherence to the DASH diet during early pregnancy was not associated with any pregnancy outcomes or complications, including hypertensive disorders of pregnancy [57]. This suggests that timing of dietary intervention, population characteristics, or other factors may influence the effectiveness of the DASH diet in preventing hypertensive disorders.

Beyond hypertensive disorders, DASH diet adherence appears to influence multiple pregnancy and neonatal outcomes through various physiological mechanisms. Table 3 consolidates findings related to secondary outcomes across studies, highlighting the broader potential benefits of this dietary pattern during pregnancy. These outcomes include favorable effects on birth weight, placental function, metabolic parameters, and various maternal health indicators, demonstrating the multifaceted impact of maternal diet quality.

### 3.4. DASH Diet and Metabolic Parameters in Pregnancy

Belfort et al. reported that while the DASH diet did not significantly affect preeclampsia incidence, it positively influenced oxidative stress markers [44]. Glutathione peroxidase levels significantly increased in the DASH diet group (intra-group analysis; mean difference = 1588 [CI 181, 2994], *p* = 0.03) and tended to differ from the variation in the standard diet group (*p* = 0.09) [44]. Glycated hemoglobin levels decreased significantly and similarly in both standard diet group (−0.61 [CI −0.96, −0.26], (*p* < 0.001) and DASH diet group (−1.1 [CI −0.57, −1.62] (−1.6, −0,6), *p* < 0.001) [50].

### 3.5. DASH Diet in Different Populations

Miller et al. examined the association between diet quality and the development of hypertensive disorders of pregnancy in an Asian and Pacific Islander cohort of 55 participants. Across gestation, participants who did not develop hypertensive disorders had better diet quality than those who did. Every point higher on the DASH diet score was associated with approximately 30% reduced odds of developing hypertensive disorders [58].

The efficacy of the DASH diet appears to differ across populations. Wiertsema et al.’s study in Dutch women found more modest effects compared to other studies, suggesting that baseline dietary patterns and population-specific factors might influence the magnitude of benefits observed [53]. This contrasts with the more pronounced effects seen in the Asian and Pacific Islander cohort studied by Miller et al. [58] and in the intervention studies by Najafian et al. [55] and Jiang et al. [56].

### 3.6. Sodium Intake and Hypertensive Disorders

An important aspect of the DASH diet is its sodium regulation. Using Danish National Birth Cohort data, Arvizu et al. found that sodium intake during pregnancy is positively related to the risk of hypertensive disorders [59]. Women with increased sodium intake (median 3.70 g/d) had 54% (95% CI: 16%, 104%) higher risk of gestational hypertension and 20% (95% CI: 1%, 42%) higher risk of preeclampsia than women with the lowest intake (median 2.60 g/d) [53]. This highlights that the sodium reduction component of the DASH diet may be particularly important for preventing hypertensive disorders of pregnancy.

### 3.7. Long-Term Health Implications

The DASH diet may have implications for long-term cardiovascular health in women with a history of hypertensive disorders of pregnancy. Timpka et al. examined lifestyle factors in the progression from hypertensive disorders to chronic hypertension using data from the Nurses’ Health Study II. The study found that being overweight or obese was the only lifestyle factor consistently associated with a higher risk of chronic hypertension, emphasizing the importance of maintaining a healthy weight for women with a history of hypertensive disorders of pregnancy [60].

### 3.8. DASH Diet Adherence and Genotypic Considerations

Courtney et al. reported that pregnant women in their cohort had moderate adherence to the DASH diet, with mean (SD) DASH scores in trimesters 1, 2, and 3 of 26.7 (5.2), 26.7 (5.3), and 26.8 (5.2), respectively. A higher DASH score was linked to several health-supporting behaviors, including taking micronutrient supplements, not smoking, maintaining a healthy BMI, being older, achieving appropriate weight gain and higher education [52].

Santos et al. [54] investigated potential genotypic influences on the response to the DASH diet by analyzing the effect of FTO (rs9939609, rs17817449) and ADRB2 (rs1042713, rs1042714) polymorphisms. No significant genotypic effect on the development of hypertensive disorders of pregnancy was found, nor any significant diet–gene interaction. A significant portion of participants adhered well to their assigned diet at the visit closest to delivery. Specifically, 39.5% of those following the traditional diet and 40.7% of participants on the DASH diet maintained high dietary compliance [54].

Similarly, in Wiertsema et al.’s Dutch cohort, the mean DASH score was 24.6 (SD 4.6), with women having higher DASH scores being more likely to be older, nulliparous, have lower pre-pregnancy BMI, higher education, lower smoking rates, and higher folic acid supplement use [53]. This suggests that adherence to the DASH diet correlates with other health-promoting behaviors in pregnancy, which may influence the relationship between diet and pregnancy outcomes.

## 4. Discussion

The findings from this literature review suggest that the DASH diet has significant potential as a preventive strategy for preeclampsia and other hypertensive disorders of pregnancy. The evidence indicates several mechanisms through which the DASH diet may confer protective effects, including improved maternal blood pressure control, enhanced endothelial function, reduced oxidative stress, and improved metabolic parameters.

### 4.1. Interpretation of Key Findings

The available evidence demonstrates that higher adherence to the DASH dietary pattern is associated with a reduced risk of preeclampsia in observational studies [48,49]. The two large prospective cohort studies by Arvizu et al. [48] and Cao et al. [49] consistently reported significant risk reductions (35% and 45%, respectively) for preeclampsia among women with the highest DASH diet adherence compared to those with the lowest adherence. These findings align with the systematic review by Li et al., which found that following the DASH diet during pregnancy decreased the risk of gestational preeclampsia (RR = 0.667; 95% CI: 0.451, 0.987) [61].

Intervention studies, although fewer in number and typically with smaller sample sizes, have shown promising results. The randomized controlled trials by Jiang et al. [56] and Najafian et al. [55] both demonstrated significant reductions in preeclampsia incidence among women following the DASH diet. However, other trials such as those by Belfort et al. [50] and Vesco et al. [51] found non-significant trends toward benefit, suggesting that factors such as study population characteristics, timing of intervention, and intervention duration may influence outcomes.

The DASH diet’s effects on blood pressure during pregnancy are particularly noteworthy. Significant reductions in diastolic blood pressure have been demonstrated with higher DASH diet adherence [52,53]. Given that hypertension is the defining feature of preeclampsia, these blood pressure-lowering effects may be a primary mechanism through which the DASH diet reduces preeclampsia risk. This is consistent with findings from non-pregnant populations, where the DASH diet has been consistently shown to lower blood pressure levels [62,63,64].

The beneficial effects extend beyond blood pressure control to include other pregnancy outcomes. The meta-analysis by Li et al. found significant reductions in the risk of fetal macrosomia (RR = 0.294; 95% CI: 0.120, 0.721) and large-for-gestational-age births (RR = 0.452; 95% CI: 0.211, 0.969) with DASH diet interventions [61]. These findings are clinically relevant, as macrosomia and being large for gestational age are associated with increased risks of cesarean delivery, shoulder dystocia, and other adverse neonatal outcomes [65,66].

Additionally, the DASH diet has shown positive effects on glycemic parameters, with a significant reduction in fasting plasma glucose levels (WMD = −6.239 mg/dL; 95% CI: −11.915, −0.563) reported in the meta-analysis [55]. This glycemic control benefit may be particularly relevant for women with gestational diabetes, who face an increased risk of developing preeclampsia [67].

### 4.2. Mechanisms of Action

Multiple physiological pathways potentially mediate DASH dietary protection against preeclamptic development. The antioxidant-rich profile of DASH nutritional components may counteract oxidative stress cascade involvement in preeclamptic pathogenesis through enhanced cellular protection mechanisms [35,36,68]. Oxidative stress plays a central role in placental dysfunction and the subsequent maternal inflammatory response characteristic of preeclampsia [69]. The antioxidant compounds abundant in the DASH diet, including vitamins C and E, polyphenols, flavonoids, and carotenoids [29,30], may help neutralize reactive oxygen species and protect against oxidative damage [68]. This is supported by Belfort et al.’s finding that the DASH diet increased glutathione peroxidase levels, an important antioxidant enzyme, in pregnant women with diabetes [50].

A critical mechanism that has received increasing attention involves the DASH diet’s effect on homocysteine metabolism [38,39]. The diet’s rich content of folate (from vegetables and fruits), vitamin B6 (from whole grains and lean proteins), and vitamin B12 (from dairy products and lean meats) may help regulate homocysteine metabolism [38,39]. Elevated homocysteine levels have been associated with increased preeclampsia risk through mechanisms involving endothelial dysfunction, oxidative stress, and thrombosis [40,41]. Studies have demonstrated that hyperhomocysteinemia is present in women with preeclampsia, with significantly higher levels compared to normotensive pregnant women [40,41]. The B-vitamins provided by the DASH diet serve as essential cofactors in homocysteine metabolism, facilitating its conversion to methionine and cysteine through remethylation and transsulfuration pathways [38]. Meta-analyses have shown that adequate B-vitamin intake can reduce homocysteine levels by 20–30%, potentially translating to significant reductions in vascular risk [42,43]. By providing adequate B-vitamins, the DASH diet may help maintain normal homocysteine levels and reduce associated vascular risks, representing an important pathway through which dietary intervention can influence preeclampsia development.

The DASH diet is also characterized by a higher intake of minerals such as calcium, magnesium, and potassium, which play crucial roles in vascular function and blood pressure regulation [33,34]. Calcium, in particular, has been extensively studied in relation to preeclampsia prevention. A meta-analysis of calcium supplementation trials showed a significant reduction in preeclampsia risk, particularly among women with low calcium intake [70]. Calcium influences vascular smooth muscle function and may reduce parathyroid hormone release, which in turn decreases intracellular calcium in vascular smooth muscle and promotes vasodilation [34]. Magnesium, abundant in the nuts, legumes, and whole grains emphasized in the DASH diet, acts as a natural calcium channel blocker and may contribute to improved vascular reactivity [33]. Potassium, found in fruits, vegetables, and legumes, helps maintain fluid balance and may counteract the effects of sodium on blood pressure [34].

The anti-inflammatory properties of the DASH diet may also contribute to its protective effects against preeclampsia. Systematic reviews have found that the DASH diet reduces inflammatory markers such as C-reactive protein in non-pregnant populations [71,72]. The anti-inflammatory effects may be attributed to both the beneficial components included in the diet (omega-3 fatty acids, antioxidants, fiber) and the reduced intake of pro-inflammatory substances (saturated fats, refined carbohydrates) [72]. Given that preeclampsia is characterized by an exaggerated inflammatory response, with elevated levels of pro-inflammatory cytokines and immune cell activation, the anti-inflammatory effects of the DASH diet may help mitigate this pathophysiological process [69,73].

The DASH diet’s emphasis on whole grains and limitations on refined carbohydrates may lead to improved insulin sensitivity and a lower glycemic response [74]. Studies show that insulin resistance contributes to preeclampsia pathogenesis through endothelial dysfunction, increased sympathetic nervous system activity, and sodium retention, with affected women demonstrating higher insulin resistance than those with normotensive pregnancies [75]. By improving insulin sensitivity through its low-glycemic index carbohydrate sources and balanced macronutrient composition, the DASH diet may help prevent these pathophysiological changes [74].

Additionally, the DASH diet may influence angiogenic balance, which is disrupted in preeclampsia. Preeclampsia is characterized by an imbalance between pro-angiogenic factors (such as placental growth factor, PlGF) and anti-angiogenic factors (such as soluble fms-like tyrosine kinase-1, sFlt-1) [12,13]. While limited research has directly examined the effects of the DASH diet on these angiogenic factors during pregnancy, nutritional components of the diet, such as polyphenols and omega-3 fatty acids, have been shown to promote angiogenesis and improve endothelial function in non-pregnant populations [76].

The dietary nitrates present in leafy greens and other vegetables emphasized in the DASH diet may also contribute to its blood pressure-lowering effects through the nitric oxide pathway [76]. Nitric oxide is a potent vasodilator and plays a crucial role in maintaining vascular homeostasis during pregnancy. Impaired nitric oxide production has been implicated in the pathophysiology of preeclampsia, contributing to endothelial dysfunction and increased vascular resistance [69]. The dietary nitrates in the DASH diet can be converted to nitric oxide through both enzymatic and non-enzymatic pathways, potentially enhancing vasodilation and improving placental blood flow [76]. Multiple studies in our review documented improved vascular function among women with higher DASH diet adherence [52,53]. Key dietary components—potassium, magnesium, and dietary nitrates from vegetables—have been shown to enhance endothelial function [44,45,46,47]. Since endothelial dysfunction constitutes a fundamental pathophysiological mechanism underlying preeclampsia development [11,47,69], these vascular protective properties likely represent a primary pathway through which the DASH diet confers its preventive effects.

An important aspect of the DASH diet’s protective mechanism involves sodium regulation. Using Danish National Birth Cohort data, our review identified that sodium intake during pregnancy is positively related to the risk of hypertensive disorders [59]. Women with an increased sodium intake had a 54% higher risk of gestational hypertension and a 20% higher risk of preeclampsia than women with the lowest intake [59]. The DASH diet’s sodium restriction component (2300 mg per day in the standard DASH diet, 1500 mg per day in the low-sodium DASH diet) [28,31] may be particularly important for preventing hypertensive disorders of pregnancy by reducing fluid retention and vascular resistance.

**Figure 1:**
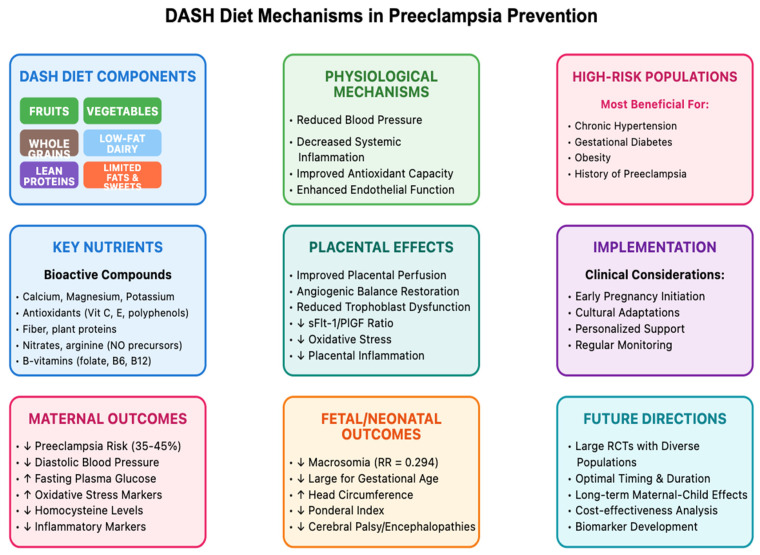
DASH diet & mechanisms in preeclampsia prevention.

Figure 1 presents a comprehensive model of the DASH diet’s effects on preeclampsia prevention, illustrating the pathways from dietary components through physiological mechanisms to maternal and fetal outcomes. As depicted, the DASH diet’s nutrient profile influences multiple biological systems that collectively contribute to reduced preeclampsia risk.

### 4.3. Comparison with Other Dietary Interventions

The DASH diet shares several features with the Mediterranean diet, another dietary pattern that has shown promising results in reducing pregnancy complications [24,25,26,77]. Both diets emphasize fruits, vegetables, whole grains, and moderate consumption of lean proteins, while limiting saturated fats and processed foods. A systematic review by Renzo et al. found that higher adherence to the Mediterranean diet was associated with a lower risk of preeclampsia and other adverse pregnancy outcomes [78]. This suggests that multiple dietary patterns that prioritize plant-based, nutrient-dense foods may confer protective effects against preeclampsia.

Comparing the DASH and Mediterranean diets reveals both similarities and differences that may influence their effectiveness in preeclampsia prevention. While both emphasize fruits, vegetables, and whole grains, the DASH diet places greater emphasis on low-fat dairy products, which are rich sources of calcium and vitamin D [76]. The Mediterranean diet, on the other hand, places more emphasis on olive oil as the primary source of fat, which is rich in monounsaturated fatty acids and polyphenols with anti-inflammatory properties [77]. Both dietary patterns limit red meat and processed foods, but the Mediterranean diet includes moderate consumption of fish, which provides omega-3 fatty acids that may have beneficial effects on placental function and inflammatory processes [79].

Low-carbohydrate and low-glycemic index diets have also been studied in relation to pregnancy outcomes [80]. While these diets may improve glycemic control in pregnant women with gestational diabetes, they do not consistently show the same range of benefits on blood pressure and preeclampsia risk as the DASH diet [81]. This suggests that the balanced approach of the DASH diet, with its emphasis on multiple food groups and nutrients rather than restriction of a single nutrient, may be more effective for preeclampsia prevention.

Vegetarian and plant-based diets have also been examined for their effects on pregnancy outcomes [26]. These diets share some features with the DASH diet, particularly the high intake of fruits, vegetables, and whole grains [19,20,23,24]. Studies suggest that vegetarian diets may be associated with lower blood pressure during pregnancy, although specific data on preeclampsia prevention is limited [26]. However, vegetarian diets may pose challenges for meeting certain nutrient requirements during pregnancy, particularly iron, vitamin B12, and omega-3 fatty acids, which are important for fetal development [26,82]. The DASH diet, with its inclusion of lean meats, fish, and dairy products, may offer a more balanced approach to meeting these nutritional needs while still providing blood pressure-lowering benefits [29,30,31].

Energy-restricted diets have been studied primarily in the context of gestational weight gain management, particularly among overweight and obese pregnant women [77]. While excessive gestational weight gain is a risk factor for preeclampsia, energy restriction alone does not address the specific nutritional factors that may influence preeclampsia pathophysiology [83,84]. The DASH diet, in contrast, focuses on dietary quality rather than caloric restriction, providing essential nutrients while naturally supporting appropriate weight gain through its emphasis on nutrient-dense, fiber-rich foods [76].

### 4.4. DASH Diet Adherence and Implementation Strategies

The effectiveness of the DASH diet in preventing preeclampsia depends on consistent adherence throughout pregnancy, which poses both challenges and opportunities for clinical implementation. Studies have reported variable levels of adherence to the DASH diet during pregnancy, with factors such as socioeconomic status, cultural food preferences, food accessibility, and nutrition knowledge influencing adherence patterns [52,85].

Courtney et al. reported that pregnant women in their cohort had moderate adherence to the DASH diet, with mean DASH scores in trimesters 1, 2, and 3 of 26.7 (out of a possible 40), indicating room for improvement. Higher DASH scores were associated with several health-supporting behaviors, including taking micronutrient supplements, not smoking, maintaining a healthy BMI, being older, achieving appropriate weight gain, and higher education [52]. This suggests that DASH diet adherence may cluster with other positive health behaviors, reflecting a broader pattern of health consciousness that could influence pregnancy outcomes.

Implementation strategies that have shown promise in promoting adherence to the DASH diet during pregnancy include individualized dietary counseling, group education sessions, regular monitoring and feedback, practical tools like meal plans and recipes, and involvement of family members in dietary changes [26,86,87]. The DASH diet’s flexibility and compatibility with various food preferences and cultural traditions make it adaptable to diverse population groups, an important consideration in the global context of preeclampsia prevention [85].

Cost considerations may also influence adherence, as the fresh fruits, vegetables, and lean proteins emphasized in the DASH diet can be more expensive than processed and energy-dense foods [85]. Strategies to address cost barriers include emphasizing seasonally available produce, incorporating frozen fruits and vegetables, utilizing legumes as economical protein sources, and connecting pregnant women with food assistance programs when applicable [87].

Technology-based approaches, including smartphone applications, web-based platforms, and telehealth interventions, have shown promise in supporting dietary adherence during pregnancy [86,87]. These approaches can provide real-time guidance, personalized feedback, and convenient tracking of dietary intake, potentially increasing engagement with dietary recommendations [87]. The study by Van Horn et al. utilized technology-enhanced methods to support adherence to a modified DASH diet (MAMA-DASH) among overweight and obese pregnant women, demonstrating the feasibility of this approach [17].

Cultural adaptations of the DASH diet may be necessary to ensure acceptability and sustainability across diverse populations [26,85]. For example, adaptations might include incorporating traditional grain varieties, culturally familiar vegetables and protein sources, and cooking methods that align with cultural preferences while maintaining the core principles of the DASH dietary pattern [26,85]. Such adaptations should be developed in consultation with members of the target cultural group and nutrition professionals familiar with both the DASH diet principles and the specific cultural context.

### 4.5. Population-Specific Considerations

The effectiveness of the DASH diet for preeclampsia prevention may vary across different population groups, influenced by factors such as pre-pregnancy BMI, age, parity, ethnicity, pre-existing medical conditions, and socioeconomic status. Understanding these population-specific considerations is crucial for tailoring preventive strategies and identifying those who might benefit most from DASH diet interventions [3,88,89].

Women with pre-existing hypertension represent a high-risk group for developing preeclampsia, with rates up to 25% compared to 3–5% in the general pregnant population [69,89,90]. The studies by Jiang et al. [56] and Najafian et al. [55] specifically focused on this high-risk population and found significant reductions in preeclampsia incidence with DASH diet interventions. This suggests that women with chronic hypertension may be a particularly appropriate target group for DASH diet recommendations [69,90,91]. The International Society for the Study of Hypertension in Pregnancy guidelines emphasize the importance of targeted interventions for high-risk women, supporting the rationale for implementing DASH diet strategies in this population [3].

Obesity is another major risk factor for preeclampsia, with obese women having a two- to three-fold increased risk compared to normal-weight women [60,92]. The relationship between obesity and preeclampsia involves multiple mechanisms, including chronic inflammation, insulin resistance, oxidative stress, and lipid abnormalities [84,92]. The DASH diet may address several of these pathophysiological mechanisms through its anti-inflammatory properties, positive effects on insulin sensitivity, and improvement in lipid profiles [61,68]. However, the study by Vesco et al. found that while the DASH diet combined with physical activity and weight management improved gestational weight gain patterns in obese women, it did not significantly reduce preeclampsia risk [51]. This suggests that additional interventions may be needed for obese pregnant women, or that timing and intensity of the intervention may be critical factors in this population [51,83]. The LIMIT randomized trial by Dodd et al. provides additional context for lifestyle interventions in overweight and obese pregnant women, highlighting the complexity of managing this high-risk group [83].

Gestational diabetes mellitus (GDM) increases the risk of preeclampsia by two to four fold, with shared pathophysiological mechanisms including insulin resistance, endothelial dysfunction, and inflammation [67]. The studies by Asemi et al. [9,11] and Yao et al. [2] demonstrated that the DASH diet improved glycemic control and reduced preeclampsia-related outcomes in women with GDM. These findings suggest that the DASH diet may be particularly beneficial for women with GDM or those at high risk of developing it [61,67].

Ethnic and racial disparities in preeclampsia risk are well documented, with higher rates observed among African American, Hispanic, and some Asian populations compared to white women. These disparities persist even after adjusting for socioeconomic factors and medical comorbidities, suggesting potential genetic, epigenetic, or cultural influences [5,6,69]. Limited research has specifically examined the effectiveness of the DASH diet across different ethnic groups during pregnancy. However, Miller et al.’s study in an Asian and Pacific Islander cohort found that higher DASH diet scores were associated with reduced odds of developing hypertensive disorders of pregnancy [58]. This suggests potential benefits across diverse ethnic groups, although more research is needed to establish population-specific effectiveness and potential adaptations needed for various cultural contexts [85]. The global burden of maternal mortality from preeclampsia, as documented by Ghulmiyyah and Sibai, and the regional variations in eclampsia burden highlighted by Giordano et al., underscore the importance of culturally appropriate interventions [5,6].

Socioeconomic status significantly influences both dietary patterns and preeclampsia risk, with lower socioeconomic status associated with poorer diet quality and higher preeclampsia rates [84]. Barriers to adopting the DASH diet among lower-income populations may include limited access to fresh foods, financial constraints, time limitations for food preparation, and lower nutrition literacy [85]. Interventions targeting these populations may need to address structural barriers to healthy eating, such as food availability and affordability, while providing practical, culturally appropriate guidance on implementing DASH principles within existing constraints [85,87]. The work by Lucas et al. on nutrition advice during pregnancy highlights the challenges healthcare professionals face in providing effective dietary guidance, particularly for disadvantaged populations [85]. The personalized intervention approach studied by Jing et al. demonstrates potential strategies for overcoming some of these barriers through tailored support [87]. Additionally, the dietary patterns observed in obese pregnant women, as documented by Flynn et al., illustrate the complex interplay between socioeconomic factors and dietary behaviors that must be considered when implementing DASH diet interventions [86].

The long-term implications of these population-specific considerations extend beyond pregnancy outcomes. Timpka et al.’s analysis of lifestyle factors in the progression from hypertensive disorders to chronic hypertension emphasizes that certain populations may face greater long-term cardiovascular risks, making targeted interventions during pregnancy even more critical [60]. This longitudinal perspective reinforces the importance of tailoring DASH diet recommendations to specific population needs and characteristics to maximize both immediate and long-term health benefits.

### 4.6. Integration with Other Preventive Strategies

The DASH diet should be considered as one component of a comprehensive approach to preeclampsia prevention, potentially complementing other established preventive strategies such as low-dose aspirin, calcium supplementation, and lifestyle modifications including physical activity and stress management [70,91,93].

Low-dose aspirin (75–150 mg daily) is currently recommended for women at high risk of preeclampsia, based on substantial evidence from randomized controlled trials and meta-analyses showing risk reductions of 10–30% [16,17,91]. The mechanisms through which aspirin prevents preeclampsia include antiplatelet effects, improved placental perfusion, and modulation of inflammatory pathways [16]. The DASH diet could potentially enhance these effects through its own anti-inflammatory properties and cardiovascular benefits [71,76]. While no studies have specifically examined the combined effects of the DASH diet and aspirin on preeclampsia prevention, the complementary mechanisms suggest potential synergistic benefits that warrant investigation [91].

Calcium supplementation (1000–2000 mg daily) has shown effectiveness in preventing preeclampsia, particularly among women with low calcium intake [18,70]. The DASH diet naturally provides increased calcium through its emphasis on low-fat dairy products, potentially reducing the need for high-dose supplements or enhancing their effectiveness [76]. The combined approach of the DASH diet plus targeted calcium supplementation based on individual needs may offer advantages over either strategy alone, particularly in populations with habitually low calcium intake [70].

Physical activity during pregnancy has been associated with reduced preeclampsia risk, with meta-analyses of randomized controlled trials showing risk reductions of 20–35% with regular moderate-intensity exercise. Exercise improves cardiovascular function, reduces inflammation, enhances insulin sensitivity, and promotes healthy weight gain—all factors relevant to preeclampsia prevention [93]. The study by Vesco et al. combined the DASH diet with physical activity recommendations for obese pregnant women, though the distinct contributions of each component to outcomes were not separated [51].

Stress reduction techniques show preliminary evidence for reducing pregnancy blood pressure by addressing chronic stress pathways (inflammation, sympathetic nervous system activity, HPA axis activation), suggesting that combining the DASH diet with stress management could offer a more holistic approach to preeclampsia prevention by targeting both physiological and psychological risk factors [94].

Weight management interventions for overweight and obese women before and during pregnancy represent another important preventive strategy, given the strong association between obesity and preeclampsia risk [84,92,95]. The DASH diet naturally supports appropriate weight management through its emphasis on nutrient-dense, fiber-rich foods with moderate caloric density [76]. Integrating the DASH diet within broader weight management programs that include physical activity and behavioral strategies could enhance effectiveness for overweight and obese women [83,92].

Since pathophysiological processes underlying preeclampsia begin early in pregnancy or before conception, implementing the DASH diet during preconception care could improve placentation and vascular adaptation, particularly for high-risk women with obesity, chronic hypertension, or previous preeclampsia—a benefit supported by Arvizu et al.’s finding that pre-pregnancy DASH adherence was associated with lower preeclampsia risk [48,96].

### 4.7. Clinical Implications and Practice Recommendations

This review suggests that the DASH diet should be considered as a preventive strategy for women at high risk of preeclampsia, particularly those with pre-existing hypertension, obesity, or prior preeclampsia history [89,90]. The diet aligns with general pregnancy nutrition guidelines without safety concerns, making it both pragmatic and feasible [97]. Implementation should begin early—ideally during preconception counseling—to allow gradual adoption and maximize benefits for placentation and early pregnancy adaptations [91]. Practical guidance should include both general DASH principles and personalized meal planning strategies that respect individual preferences and cultural considerations [79].

For pregnant women, standard DASH recommendations require adaptation to meet increased nutrient needs. These modifications should include additional calcium-rich foods (1000–1300 mg/day), iron-rich foods to prevent anemia, and adequate omega-3 fatty acids for fetal neurodevelopment [97]. The modified pregnancy DASH diet maintains emphasis on fruits, vegetables, whole grains, and low-fat dairy while ensuring adequate protein, essential fatty acids, and micronutrients [9,17,57]. Maintaining adherence requires regular dietary assessment using simplified scoring systems, collaborative goal-setting, problem-solving for barriers, and positive reinforcement [86,87].

Effective DASH diet implementation requires integration into existing prenatal care through routine assessments and dietitian referrals, multidisciplinary collaboration among healthcare providers, and addressing equity concerns by providing economical food guidance, connecting women with assistance programs, and advocating for improved healthy food access in underserved communities [85,87].

### 4.8. Limitations of Current Evidence

Despite promising findings, the evidence base for the success of the DASH diet in preeclampsia prevention has significant limitations. Randomized controlled trials are few, typically involving small sample sizes and methodological constraints [98], limiting definitive conclusions about efficacy. Study heterogeneity—including diverse populations (women with gestational diabetes, chronic hypertension, and obesity), varying intervention protocols, and inconsistent outcome measures—makes direct comparisons challenging [61]. The DASH interventions themselves have varied in duration, intensity, and specific dietary components, obscuring the identification of optimal approaches [61,98].

Most studies rely on self-reported dietary assessments through food frequency questionnaires, introducing recall bias and measurement errors [99], while few utilize objective biomarkers like urinary sodium excretion or plasma antioxidant levels to confirm adherence [100]. Intervention timing varies considerably, with some studies focusing on preconception or early pregnancy [48,57] and others implementing interventions in later trimesters [55,56], leaving uncertainty about optimal timing despite evidence that preeclampsia pathophysiology begins early in pregnancy [7,11]. There is also limited information on effect modifiers such as genetic factors, pre-pregnancy dietary patterns, and concurrent lifestyle factors that may influence intervention effectiveness [54,101].

The long-term effects of DASH diet adherence during pregnancy remain largely unexplored. While studies document short-term benefits during pregnancy and immediate postpartum periods, longitudinal research examining impacts on maternal cardiovascular health and offspring development is scarce [83,96]. This gap is particularly significant given the established link between preeclampsia and future cardiovascular disease risk [96]. Understanding these potential long-term protective effects represents an important area for future research that could strengthen the case for DASH diet implementation in clinical practice and public health interventions targeting high-risk pregnant women.

### 4.9. Future Research Directions

Despite promising findings, several critical research gaps need addressing to establish the DASH diet’s role in preeclampsia prevention. Large-scale, multicenter randomized controlled trials with diverse populations are urgently needed to definitively determine efficacy, optimal timing, and dose–response relationships [102]. These trials should examine implementation across various clinical settings and risk profiles, particularly investigating when intervention would be most effective during the reproductive timeline (preconception, early pregnancy, or later) [94,96]. Mechanistic studies exploring the biological pathways through which the DASH diet influences placental development, angiogenic balance, and inflammation would enhance the understanding of its protective effects and could identify the most beneficial dietary components [92,101].

Additionally, research examining potential synergies between the DASH diet and other preventive strategies (including aspirin, calcium supplementation, and physical activity) would help develop comprehensive approaches to preeclampsia prevention [91,93]. Longitudinal studies assessing long-term maternal cardiovascular outcomes and offspring development following DASH diet adherence during pregnancy would provide valuable insights into intergenerational health implications [96,103]. Implementation science research investigating practical strategies for promoting adherence across diverse sociocultural contexts and resource settings is also essential for translating evidence into clinical practice and public health impact [94,100].

## 5. Conclusions

This literature review provides compelling evidence that the DASH diet may be an effective strategy for preeclampsia prevention. The diet’s combination of fruits, vegetables, whole grains, low-fat dairy, and limited saturated fats appears to reduce the risk of preeclampsia through multiple mechanisms, including improved blood pressure control, enhanced endothelial function, reduced oxidative stress, and improved metabolic parameters. Observational studies consistently show 35–45% risk reductions among women with high DASH adherence, while intervention trials in high-risk populations demonstrate promising results, particularly for women with pre-existing hypertension and gestational diabetes. Despite limitations in the current evidence base, the DASH diet represents a safe, feasible approach that aligns with general pregnancy nutrition recommendations and could be readily integrated into prenatal care, with appropriate adaptations for individual needs and cultural contexts. Future research should address optimal timing, population-specific effectiveness, and potential synergies with other preventive strategies to strengthen the evidence base for this promising nutritional approach to reducing the burden of preeclampsia.

## Figures and Tables

**Table 1:** Studies on the DASH diet and preeclampsia prevention: key characteristics and findings.

First Author (Year)	Country	Study Design	Sample Size and Characteristics	DASH Diet Assessment Method	Study Duration/Follow-Up	Key Outcomes Measured	Key Findings	Confounders Controlled
Arvizu et al. (2020) [48]	USA	Prospective cohort	16,892 singleton pregnancies among 11,535 women from the Nurses’ Health Study II	DASH score based on food frequency questionnaire evaluating intake of food groups	Pre-pregnancy dietary patterns and subsequent pregnancies	Preeclampsia risk	Women in the highest quintile of DASH score had a 35% lower risk of preeclampsia compared to those in the lowest quintile (RR: 0.65; 95% CI: 0.48, 0.87; *p*-trend = 0.01)	Age, BMI, parity, family history, smoking, physical activity, energy intake
Cao et al. (2020) [49]	China	Case-control	449 preeclampsia cases and 449 controls	DASH-style diet score based on food frequency questionnaire	Prenatal diet evaluated during the last three months of pregnancy	Preeclampsia risk	Participants in the fourth quartile of DASH score were 45% less likely to have preeclampsia than those in the first quartile (adjusted OR: 0.53; 95% CI: 0.36, 0.78; *p*-trend = 0.001)	Age, BMI, education, physical activity, parity, family history of hypertension, energy intake, GDM (Gestational Diabetes Mellitus)
Belfort et al. (2023) [50]	Brazil	Randomized controlled trial	87 women with pre-existing diabetes mellitus undergoing pregnancy	DASH diet intervention vs. standard diet	Nutrition intervention from enrollment till delivery (18 weeks)	Preeclampsia incidence, oxidative stress markers	No statistically significant difference in preeclampsia incidence between groups (22.9% in standard diet vs. 12.1% in DASH diet group, *p* = 0.25); Glutathione peroxidase levels significantly increased in DASH diet group	Maternal age, gestational age, BMI, glycated hemoglobin
Vesco et al. (2014) [51]	USA	Randomized controlled trial	114 obese women (56 intervention, 58 control)	DASH diet with sodium restriction plus moderate physical activity vs. standard care	Throughout pregnancy until delivery	Gestational weight gain, preeclampsia incidence	Preeclampsia incidence: 9% in intervention group vs. 10% in control group (*p* = 0.101) (OR: 0.85; 95% CI: 0.24–2.96); Intervention group gained less weight during pregnancy	Maternal age, pre-pregnancy BMI, parity, race/ethnicity, smoking, baseline blood pressure
Courtney et al. (2020) [52]	Ireland	Observational study	511 women from the ROLO study in the National Maternity Hospital	DASH score based on food frequency questionnaire	Throughout pregnancy with assessments in each trimester	Maternal blood pressure	Higher DASH score associated with lower diastolic blood pressure in trimesters 1 and 3; For each 5-unit increase in DASH score, 0.8 mmHg lower DBP in trimester 1 (*p* = 0.001) and 0.76 mmHg lower DBP in trimester 3 (*p* = 0.05)	Maternal age, BMI, education, parity, smoking, physical activity, energy intakes
Wiertsema et al. (2021) [53]	Netherlands	Population-based cohort	3414 Dutch women	DASH score	Throughout pregnancy with measurements in each trimester	Blood pressure patterns, placental hemodynamics, gestational hypertensive disorders	Lower DASH score quartiles associated with higher mid-pregnancy diastolic blood pressure; Higher DASH score associated with lower mid-pregnancy diastolic blood pressure (−0.45 [95% CI: −0.78 to −0.12] mm Hg per SD increase); Lower DASH quartiles associated with higher mid- and late-pregnancy umbilical artery pulsatility index	Maternal age, gestational age, parity, pre-pregnancy BMI, education, ethnicity, smoking, folic acid supplement use, energy intake
dos Santos et al. (2023) [54]	Brazil	Nutrigenetic trial, part of the RCT DASDIA (DASH diet for pregnant women with Diabetes),	70 pregnant women with pregestational diabetes mellitus (*n* = 29 DASH diet group vs. *n* = 41 traditional diet group)	Traditional vs. DASH diet groups	Throughout pregnancy	Blood pressure trajectory	No significant differences in blood pressure trajectory between diet groups; Both groups showed normal physiological patterns with increases only after 32.5 weeks	Age, genetic polymorphisms (FTO and ADRB2), pregestational BMI, DM type, chronic diseases, educational and marital status, ethnicity
Najafian et al. (2023) [55]	Iran	Randomized controlled trial	60 pregnant women with gestational or chronic hypertension	DASH diet vs. control diet	1–2 months intervention, follow up until delivery	Blood pressure, preeclampsia incidence, pregnancy outcomes (birth weight and Apgar score)	After intervention, systolic and diastolic blood pressure significantly lower in DASH diet group (*p* < 0.05); Lower incidence of preeclampsia (*p* = 0.035), preterm delivery (*p* = 0.020), and placental abruption (*p* = 0.007) in DASH diet group	Baseline blood pressure, gestational age, BMI, maternal age, parity
Jiang et al. (2019) [56]	China	Randomized controlled trial	85 pregnant women with gestational or chronic hypertension	DASH diet modified for pregnancy needs vs. medical nutrition therapy	12 weeks intervention, follow up until delivery	Preeclampsia incidence, pregnancy outcomes	Preeclampsia incidence: 43.2% in DASH group vs. 65.9% in control group (*p* = 0.036); Significant differences in gestational age at delivery and newborn body length	Maternal age, pre-pregnancy BMI, parity, baseline blood pressure
Fulay et al. (2018) [57]	USA	Longitudinal cohort	1760 women in Project Viva, a Boston-area longitudinal cohort	DASH diet score based on food frequency questionnaire	Early pregnancy	Pregnancy outcomes and complications	No association between DASH diet during early pregnancy and hypertensive disorders of pregnancy	Maternal age, race/ethnicity, education, parity, pre-pregnancy BMI, smoking, energy intake
Miller et al. (2024) [58]	USA	Cohort study	55 participants in an Asian and Pacific Islander cohort	DASH diet quality score	Throughout gestation	Hypertensive disorders of pregnancy	Participants who did not develop hypertensive disorders had better diet quality; Every point higher on DASH diet score associated with ~30% reduced odds of developing hypertensive disorders	Maternal age, BMI, ethnicity, parity, pre-existing conditions
Arvizu et al. (2020) [59]	Denmark	Cohort study	Danish National Birth Cohort	Sodium intake assessment	Prenatal diet during pregnancy	Hypertensive disorders of pregnancy	Women with highest sodium intake (median 3.70 g/d) had 54% (95% CI: 16%, 104%) higher risk of gestational hypertension and 20% (95% CI: 1%, 42%) higher risk of preeclampsia than women with lowest intake (median 2.60 g/d)	Maternal age, parity, socioeconomic status, smoking, alcohol consumption, physical activity
Timpka et al. (2017) [60]	USA	Observational cohort	54,588 parous female from Nurses’ Health Study II	Lifestyle factors including dietary pattern based on food frequency questionnaires	From pregnancy through follow-up (median 32 years)	Progression from hypertensive disorders of pregnancy to chronic hypertension	Being overweight or obese was the only lifestyle factor consistently associated with a higher risk of chronic hypertension after hypertensive disorders of pregnancy	Age, family history, race/ethnicity, physical activity, smoking

**Table 2:** Summary of DASH diet effects on gestational hypertensive disorders and preeclampsia.

Study (Year)	Study Design	Sample Size/Population	Key Findings Related to Hypertensive Disorders/Preeclampsia	Strength of Association
Arvizu et al. (2020) [48]	Prospective cohort	16,892 singleton pregnancies among 11,535 women	Women in highest quintile of DASH score had 35% lower preeclampsia risk compared to lowest quintile (RR: 0.65; 95% CI: 0.48, 0.87; *p*-trend = 0.01)	Strong inverse association
Cao et al. (2020) [49]	Case-control	449 preeclampsia cases and 449 controls	Fourth quartile of DASH score associated with 47% lower preeclampsia risk (adjusted OR: 0.53; 95% CI: 0.36, 0.78; *p*-trend = 0.001)	Strong inverse association
Belfort et al. (2023) [50]	Randomized controlled trial	Women with pre-existing diabetes mellitus	No statistically significant difference in preeclampsia incidence between DASH diet (12.1%) and standard diet (22.9%) groups (*p* = 0.25)	Non-significant trend toward benefit
Vesco et al. (2014) [51]	Randomized controlled trial	114 obese women	Preeclampsia rate in DASH intervention group (9%) vs. control group (10%) showed non-significant trend toward benefit (*p* = 0.101) (OR: 0.85; 95% CI: 0.24–2.96)	Non-significant trend toward benefit
Courtney et al. (2020) [52]	Observational study	511 women	Higher DASH score associated with lower diastolic blood pressure in trimesters 1 and 3; For each 5-unit increase in DASH score, 0.8 mmHg lower DBP in T1 (*p* = 0.001) and 0.76 mmHg lower DBP in T3 (*p* = 0.05)	Significant effect on blood pressure
Wiertsema et al. (2021) [53]	Population-based cohort	3414 Dutch women	Higher DASH score associated with lower mid-pregnancy diastolic blood pressure (−0.45 mm Hg per SD increase, 95% CI: −0.78 to −0.12); No associations with gestational hypertensive disorders	Significant effect on blood pressure, no effect on hypertensive disorders
dos Santos et al. (2023) [54]	Nutrigenetic trial	70 pregnant women with pregestational diabetes	No significant differences in blood pressure trajectory between DASH and traditional diet groups	No significant effect
Najafian et al. (2023) [55]	Randomized controlled trial	60 pregnant women with hypertension	DASH diet group had significantly lower incidence of preeclampsia (*p* = 0.035) and significantly lower systolic and diastolic blood pressure (*p* < 0.05)	Strong beneficial effect
Jiang et al. (2019) [56]	Randomized controlled trial	85 pregnant women with hypertension	Preeclampsia incidence: 43.2% in DASH group vs. 65.9% in control group (*p* = 0.036)	Significant beneficial effect
Fulay et al. (2018) [57]	Longitudinal cohort	1760 women	No association between DASH diet during early pregnancy and hypertensive disorders of pregnancy	No significant effect
Miller et al. (2024) [58]	Cohort study	55 Asian and Pacific Islander women	Every point higher on DASH diet score associated with approximately 30% reduced odds of developing hypertensive disorders	Significant beneficial effect
Arvizu et al. (2020) [59]	Cohort study	Danish National Birth Cohort	Higher sodium intake (DASH diet component) associated with 54% higher risk of gestational hypertension (95% CI: 16%, 104%) and 20% higher risk of preeclampsia (95% CI: 1%, 42%)	Significant effect of sodium intake
Timpka et al. (2017) [60]	Observational cohort	Nurses’ Health Study II	Being overweight/obese was the only lifestyle factor consistently associated with a higher risk of chronic hypertension after pregnancy hypertensive disorders	No direct DASH diet association measured

**Table 3:** DASH diet effects on secondary pregnancy outcomes beyond hypertensive disorders.

Outcome Category	Study (Year)	Study Design	Population	Key Findings	Statistical Significance
Maternal Blood Pressure	Courtney et al. (2020) [52]	Observational	511 women	0.8 mmHg lower DBP in T1 and 0.76 mmHg lower DBP in T3 per 5-unit increase in DASH score	Significant (*p* = 0.001 for T1, *p* = 0.05 for T3)
	Wiertsema et al. (2021) [53]	Cohort	3414 Dutch women	Lower mid-pregnancy diastolic blood pressure (−0.45 mm Hg per SD increase in DASH score)	Significant (95% CI: −0.78 to −0.12)
Birth Outcomes	Vesco et al. (2014) [51]	RCT	114 obese women	Lower proportion of LGA babies (9% vs. 26%)	Significant (OR: 0.28, 95% CI: 0.09–0.84)
	Jiang et al. (2019) [56]	RCT	85 hypertensive women	Significant differences in gestational age at delivery and newborn body length	Significant (*p* < 0.05)
	Najafian et al. (2023) [55]	RCT	60 hypertensive women	Lower incidence of preterm delivery	Significant (*p* = 0.020)
Placental Function	Wiertsema et al. (2021) [53]	Cohort	3414 Dutch women	Higher maternal DASH score associated with lower mid- and late pregnancy umbilical artery pulsatility index	Significant (*p* ≤ 0.05)
	Najafian et al. (2023) [55]	RCT	60 hypertensive women	Lower incidence of placental abruption	Significant (*p* = 0.007)
Metabolic Parameters	Belfort et al. (2023) [50]	RCT	Women with pre-existing diabetes	Increased glutathione peroxidase levels; decreased glycated hemoglobin	Significant (*p* = 0.03 for glutathione peroxidase)
DASH Diet Adherence Factors	Courtney et al. (2020) [52]	Observational	511 women	Higher DASH score linked to taking supplements, not smoking, healthy BMI, older age, appropriate weight gain, higher education	Significant associations
	Santos et al. (2023) [54]	RCT	70 diabetic women	40.7% of DASH group maintained high dietary compliance at delivery	Reported compliance rate
Long-term Implications	Timpka et al. (2017) [60]	Cohort	Nurses’ Health Study II	Overweight/obesity was key factor in progression from hypertensive disorders to chronic hypertension	Significant association
